# Intrinsic beam emittance of laser-accelerated electrons measured by x-ray spectroscopic imaging

**DOI:** 10.1038/srep24622

**Published:** 2016-04-19

**Authors:** G. Golovin, S. Banerjee, C. Liu, S. Chen, J. Zhang, B. Zhao, P. Zhang, M. Veale, M. Wilson, P. Seller, D. Umstadter

**Affiliations:** 1 Department of Physics and Astronomy, University of Nebraska, Lincoln NE 68588, USA; 2 Science and Technology Facilities Council, Rutherford Appleton Laboratory, Harwell Science & Innovation Campus, Didcot OX11 0QX, UK

## Abstract

The recent combination of ultra-intense lasers and laser-accelerated electron beams is enabling the development of a new generation of compact x-ray light sources, the coherence of which depends directly on electron beam emittance. Although the emittance of accelerated electron beams can be low, it can grow due to the effects of space charge during free-space propagation. Direct experimental measurement of this important property is complicated by micron-scale beam sizes, and the presence of intense fields at the location where space charge acts. Reported here is a novel, non-destructive, single-shot method that overcame this problem. It employed an intense laser probe pulse, and spectroscopic imaging of the inverse-Compton scattered x-rays, allowing measurement of an ultra-low value for the normalized transverse emittance, 0.15 (±0.06) π mm mrad, as well as study of its subsequent growth upon exiting the accelerator. The technique and results are critical for designing multi-stage laser-wakefield accelerators, and generating high-brightness, spatially coherent x-rays.

Interest in laser-wakefield electron accelerators^1^ (LWFA) has been principally motivated by their ultrahigh acceleration gradient, which is 1,000× greater than that of conventional (RF-based) alternatives. Advances in LWFA technology have also recently led to the development of a new generation of x-ray sources, including ones based on inverse-Compton-scattering (ICS), betatron emission, or free-electron lasing^2^. These accelerators and photon sources have the advantage of being small enough to fit in a university or hospital laboratory. Also, both types of beams, electron^3^,^4^ and x-ray^5^,^6^,^7^, are demonstrated to be energetic, narrow in bandwidth, tunable and have micron source size. Additionally, their pulse duration are either demonstrated (in the case of electrons) or expected (in the case of x-rays) to be just a few femtoseconds in duration^8^,^9^. These unique beam characteristics are creating new opportunities in basic and applied research^2^,^10^, such as (1) time-resolved diffraction^11^,^12^ for studies of ultrafast dynamics^13^, (2) nanoscale critical-dimension small-angle scattering^14^ for advanced semiconductor metrology, (3) micron-scale-resolution radiography^15^ for non-destructive evaluation, and (4) phase-contrast imaging^16^,^17^ and low dose radiology^18^ for bio-medical imaging.

The bandwidth, spatial coherence and spectral brightness of accelerator-based x-ray beams all depend critically on transverse electron-beam emittance. The high-field gradients (GeV/cm) of laser-wakefield electron accelerators should in principle result in low emittance beams. However, as the beams freely propagate in the vacuum region after acceleration, space-charge effects will cause the emittance to rapidly increase. Direct measurement of intrinsic emittance (before space charge acts) has posed a substantial challenge for several reasons^19^.

First, LWFA electron beams differ significantly from those produced by conventional methods. They have an order of magnitude larger energy and angular spread, orders of magnitude smaller source size, significantly smaller transverse emittance, shorter pulse duration, and lower shot-to-shot reproducibility. Second, because of their higher charge density, and hence space charge effects, the electron beam emittance is predicted by numerical simulations^20^,^21^ and theoretical modeling^22^ to grow rapidly as the electron beam freely propagates in vacuum away from the accelerator exit. Third, the high-intensity laser light co-exists simultaneously in the same region where the space charge acts. For these reasons, prior emittance measurements of LWFA electron beams have been unable to account for this space charge growth.

For example, the pepper-pot technique^20^,^23^,^24^,^25^ was recently shown to overestimate the emittance of electron beams that have a source size smaller than 10 μm^19^, as is the case with LWFA beams^26^. Betatron emission has also been used to estimate the emittance of laser-driven electron beams, but only inside, and averaged over, the plasma acceleration region^27^,^28^. The reported emittance is likely different from the free-space value after the electron beam exits the plasma. Likewise, quadrupole scanning^29^ is unable to measure intrinsic electron-beam emittance.

We report experimental results of a novel measurement technique that overcomes the aforementioned problems. We use spectroscopic imaging of ICS x-rays^30^,^31^,^32^ to extract electron beam signatures imprinted on the spatial and spectral characteristics of the scattered x-rays, and measure the electron beam energy, energy spread, angular divergence, and source size. These parameters were used to determine the intrinsic electron-beam transverse emittance immediately at the exit of the accelerator, and after 40 cm of propagation. The observed significant change of both the angular divergence and transverse emittance is found to be consistent with the predictions of theoretical models^22^,^33^, as well as with our own numerical simulations.

## Results

**Figure 1:**
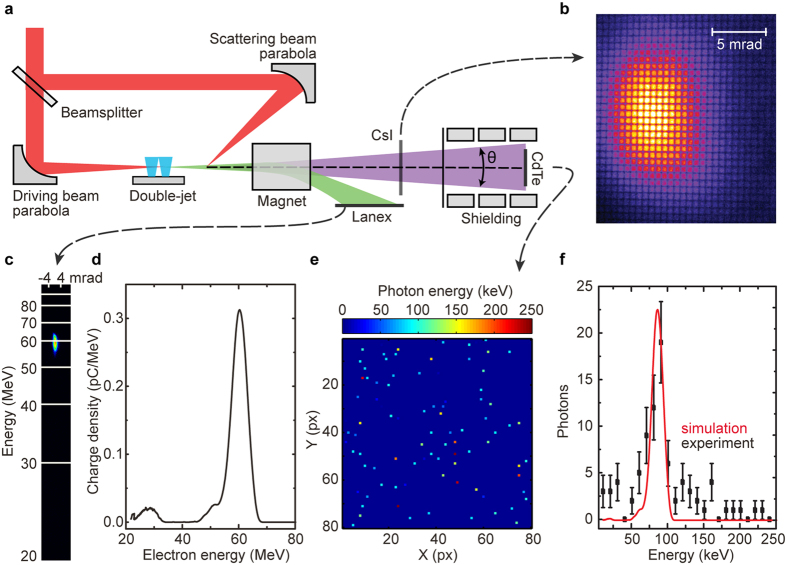
Layout of the experimental setup and a typical single-shot dataset. (**a**) Experimental setup. Spectral characteristics of an electron beam are measured using a magnetic spectrometer and a fluorescent LANEX screen. X-ray beam imaging is performed with high (0.4 mrad) angular resolution (as a function of polar angle θ) using the CdTe spectroscopic x-ray imaging detector. See Methods for additional information. (**b**) The x-ray profile measured with a CsI scintillator coupled to a 14-bit high-gain CCD. When the CdTe detector was operated, the CsI was removed from the path of the x-ray beam. (**c**) LANEX image of the dispersed electron beam. (**d**) Reconstructed electron beam spectrum based on the response function of the magnetic spectrometer. The beam has a charge of 2.4 ± 0.4 pC, central energy of 60 ± 1 MeV, and 10 ± 1% energy spread (FWHM). (**d**) Measured x-ray signal on the CdTe detector with charge sharing correction. Each dot represents a single photon event. (**f**) X-ray spectrum, measured with the CdTe detector (black, error bars represent standard deviations based on Poisson statistics), central energy is 85 ± 1 keV with 24 ± 2% energy spread (FWHM, averaged over 6-mrad cone) and simulated spectrum based on the electron beam spectrum (red). Figures (**c**–**f**) correspond to the same shot.

The experimental setup is shown in Fig. 1(a). A high-energy, ultrashort pulse from a Ti:Sapphire laser system is split in two using a beamsplitter, each part is then independently compressed. The first pulse is used to accelerate electron beams in the double-stage gas target via the process of laser-wakefield acceleration. The second pulse scatters from the electron beam at an angle of 170 deg. and generates x-rays via the process of ICS6,26. A typical x-ray beam profile, obtained from the CsI detector, is shown in Fig. 1(b). A complete set of data from all detectors, obtained in a single shot, is shown in Fig. 1(c–f). The dispersed image of the electron beam on the LANEX screen, shown in Fig. 1(c), is used to obtain the spectrum of the electron beam, shown in Fig. 1(d). The image of the x-ray beam on the CdTe detector (with charge sharing correction) for a single shot is shown in Fig. 1(e). The pulse height information is converted to energy using the measured response function of the detector. The resulting x-ray spectrum is shown in Fig. 1(f) (red curve). The black curve in Fig. 1(f) shows the simulated x-ray spectrum. Spectroscopic imaging allowed us to precisely measure the x-ray energy spread, which was found to be 24 ± 2%. This energy spread is comparable to the x-ray bandwidth from conventional RF-LINAC-based Compton sources^34^, and, to the best of our knowledge, is the narrowest yet reported from an all-laser ICS x-ray light source. Prior results reported energy spreads of 40%^5^ and 50%^6^.

**Figure 2:**
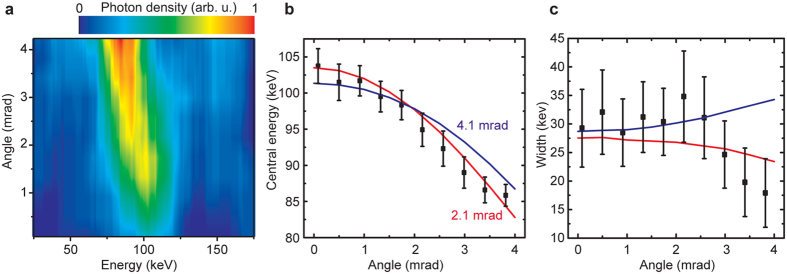
Single-shot angular-resolved x-ray spectrum measured using spectroscopic x-ray imaging. (**a**) X-ray photon spectrum as a function of polar angle. (**b**) Mean energy of the x-rays as a function of polar angle. (**c**) Dependence of the spectral width (FWHM) on the polar angle. Electron beam parameters were: central energy −65 ± 1 MeV; energy spread (FWHM) −8.9 ± 0.1 MeV; and divergence (FWHM) −4.1 ± 0.4 mrad (measured on the LANEX screen). Blue curves on (**b**,**c**) show simulated dependences based on these electron beam parameters. Red curves on (**b**,**c**) show simulated dependences based on electron beam parameters, obtained from angular-resolved x-ray spectrum: central energy −66 ± 1 MeV; energy spread (FWHM) −8.5 ± 3.5 MeV; and divergence (FWHM) −2.1 ± 0.7 mrad. Error bars represent 95% confidence bands, calculated based on least-squares fits of spectral data. Photon flux at the source is 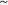
10^4^. See details on this measurement in the supplementary.

The spectroscopic x-ray imaging detector allowed us to measure angular-dependent ICS x-ray spectrum for every shot; the result is shown in Fig. 2(a). We fitted the spectra, corresponding to individual polar angles, with Gaussian functions, and extracted the resulting dependencies of central energy and energy spread on angle. The dependence of central energy of the x-rays on polar angle, shown in Fig. 2(b), arises from the dependence of the resonant scattered frequency on the polar angle (see Methods, eq. 2). The dependence of the spectral width on the polar angle, shown in Fig. 2(c), is more complex and requires accounting for both the spectral and angular characteristics of the electron beam.

The angular-resolved spectral measurements were used to determine the parameters of the electron beam. To this end, ICS simulations were performed (see Methods for details), in which three parameters of the electron beam were varied: central energy, energy spread, and divergence. The electron beam parameters could then be obtained, such that the computed and measured angular-resolved x-ray spectra coincide. For the x-ray spectrum shown in Fig. 2, we obtained the following parameters of the corresponding electron beam: central energy (66 ± 1 MeV); energy spread (8.5 ± 3.5 MeV); and divergence (2.1 ± 0.7 mrad). The angular dependence of the x-ray central energy and spread on polar angle for these electron beam parameters inferred from our model are shown in Fig. 2(b,c) as red curves. The computed parameters described the electron beam at the exit of the accelerator structure. For the same electron beam, we also measured these parameters again after 40 cm of propagation using the magnetic spectrometer and LANEX imaging screen. The measured values for the central energy and energy spread are 65 ± 1 MeV and 8.9 ± 0.1 MeV, respectively.

These experimental results show that space-charge effects do not affect the spectrum of the electron beam (on account of its high beam energy), which is found to be consistent with numerical simulations. At the same time, the electron beam divergence after 40 cm of propagation (4.1 ± 0.4 mrad) is twice what it is at the source. This increase arises from space-charge effects, as will be explained in detail later.

Figure 2(b,c) shows that performing only an on-axis measurement of ICS x-rays is not sufficient to obtain electron beam divergence at the accelerator exit. The difference between ICS x-rays generated with 2.1 and 4.1 mrad electron beams (red and blue curves, respectively) becomes pronounced only when the off-axis part of the x-ray spectrum is taken into consideration. See the supplementary section for a discussion on how on- and off-axis ICS spectral measurements affect electron beam measurement precision.

**Figure 3:**
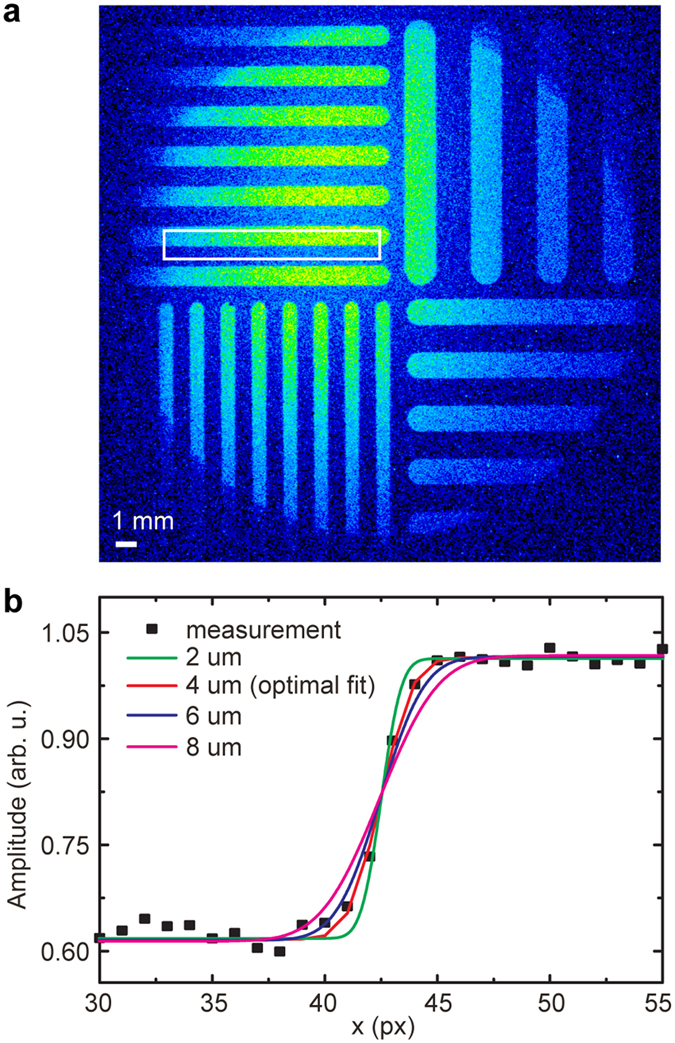
The x-ray and electron beams’ source size measurement. (**a**) Image of the test pattern (taken with an image plate). White box shows the area used for the source size analysis. (**b**) Intensity profile across the edge of the pattern (black squares) and it fits with Gauss error functions (solid lines). Red curve corresponds to an optimal fit.

Knife-edge measurements were performed to determine the source size of both the x-ray and electron beams (it should be the same for both, since the scattering laser pulse is much larger than the electron beam at the interaction point). A 2-mm thick steel pattern with different slits was placed at a distance of 2 m from the source; its shadows were imaged at a distance of 5.3 m with an image plate (30-um pixel size), yielding a magnification of 2.7. To obtain sufficiently high signal on the image plate, 100 shots were accumulated. An area with an edge shadow on the captured image (shown as a white box on Fig. 3(a)) was chosen, and its profile was plotted (see Fig. 3(b)). Each point on the profile plot shows the median of a row on the image plate picture. The use of the median improves the signal-to-noise ratio of the signal by filtering out outliers. The edge shadow profile is a convolution of the intensity distribution of the x-ray source at its origin and the edge transmission. Assuming a Gaussian intensity distribution of the source and step-transmission function of the edge, one should expect a Gauss error function as a shadow profile, with a width depending on the x-ray’s source size. After pointing fluctuation correction (2 mrad RMS), we obtained *σ*_*r*_ = 4 ± 1 um (RMS) as a final value for the source size. Such a small source size is ideal for radiography, since it permits resolution of microscopic features^15^. This also represents an improvement on the best results obtained by means of a different approach: namely, scanning the scattering laser pulse across the electron beam (5 ± 3 um RMS)^26^. Even though the presented measurement is multiple-shot (and might therefore overestimate source-size), it can be performed in a single shot given a high enough x-ray photon flux.

The emittance of the electron beam is determined by combining the source size measurement with the parameters obtained from x-ray spectroscopic imaging. The normalized electron beam transverse emittance *ε*_nt_ is computed using the formula *ε*_nt_ = *βγσ*_Θ_*σ*_*r*_, where 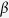 is the ratio of electron speed to the speed of light, γ is the electron beam Lorentz factor, *σ*_*θ*_ is the electron beam RMS divergence, and *σ*_*r*_ is the electron beam RMS source-size. We obtained the *ε*_nt_ = 0.15 ± 0.06 *π*mm mrad value measured at 1.5 mm from the exit of the accelerator. This value is on par with the lowest transverse emittance measured so far for an LWFA electron beam. The uncertainty can be improved by using a detector with a larger number of pixels. We also verified that the emittance value computed using this definition is consistent with the standard definition of emittance: 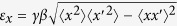. This was accomplished by reconstructing the phase space of the electron beam using measured parameters, and then computing the emittance based on the statistical definition.

Theoretical models^22^,^33^ have previously predicted emittance growth for laser-driven electron beams after they exit the accelerator and propagate in free space. Our results qualitatively agree with these predictions. In order to quantitatively determine the extent of emittance growth in our case, we performed numerical calculations that take into account the measured characteristics of the electron beam and the specific experimental geometry. The electron beam phase space was constructed using the measured energy spectrum and source size. The temporal duration was assumed to be 10 fs at the source consistent with prior measurements.

**Figure 4:**
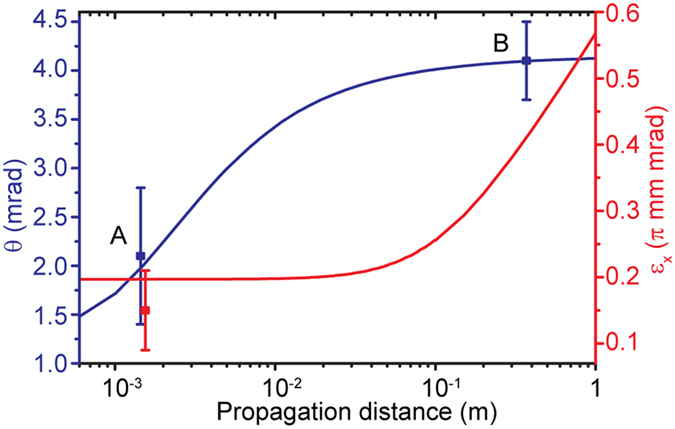
Evolution of the electron beam (divergence and normalized transverse emittance) after exit from the plasma. The divergence, calculated from the rate of change of the transverse beam size, increases rapidly over the first few mm and then reaches a limiting value while the emittance monotonically increases. Labels A and B identify locations that correspond to experimental measurements. The beam at location A is probed by the ICS technique, while the measurement at location B relies on the angular size measured on a fluorescent screen (assuming mostly straight electron trajectories). The electron beam is assumed to have an energy of 65 MeV, energy spread of 10%, and charge of 10 pC.

The evolution of the beam is calculated using a particle-tracking code based on an adaptive mesh and including the effects of space charge^35^. The measured electron beam parameters (energy, energy spread, spot-size, and divergence) fix the initial emittance of the beam. We assumed the origin to be the point where the plasma effects become negligible. Figure 4 shows the evolution of beam divergence and emittance as a function of propagation distance. The former increases rapidly over the first few mm of propagation and then reaches a limiting value. This arises from the fact that as the transverse spatial extent of the beam increases, the space-charge driven force reduces in magnitude and eventually becomes negligible. The transverse emittance, determined by both the size and angular spread of the beam, increases monotonically, and is more than doubled at 1 m compared to that at the source. In our experiments, we performed measurements of the electron beam parameters at locations A and B, the former by means of ICS, and the latter using a fluorescent screen. It is clear that the divergence growth becomes negligible beyond 3 cm. The expansion rate significantly increases for beams with higher charge or lower energy. We investigated the effects of the pulse duration on the computed results and found them to be not significant within a factor of two change in the assumed temporal duration of the electron pulse (10 fs).

Based on the measurements described previously, the beam divergence is 2.1 ± 0.7 mrad at 1.5 mm from the source (measured using single-shot spectroscopic x-ray imaging) and 4.1 ± 0.4 mrad on a fluorescent screen located at 0.37 m from the source. By comparing these values to that shown in Fig. 4, it is clear that there is excellent agreement between the measured and calculated divergence. The normalized transverse emittance determined using *ε*_nt_ = *βγσ*_*θ*_*σ*_*r*_ is dependent on location, and is significantly overestimated for measurements performed at a large distance from the source. Such limitations would be applicable to measurements performed using the pepper-pot method. It should be noted that our measurement might have slightly overestimated the value of emittance, since the electron beam was still allowed to propagate a fraction of a mm after exiting the down-ramp, before it was overlapped with the scattering laser beam. However, the simulations show that this effect is negligible (notice no change in emittance over the first few cm of propagation, red curve in Fig. 4). Also, the measured beam divergence might differ from the one inside the plasma, because of beam focusing on the plasma down-ramp^29^.

The measured value of the LWFA electron beam emittance we report here is lower than most of those previously reported in part because the measurement is performed at the source before the emittance degrades. Previous measurements were made with the pepper-pot technique^20^,^23^,^24^,^25^ and the quadrupole-scan method^29^ (even though the latter measurement was performed in a regime where space-charge effects were negligible, which is not true in our case). The transverse emittance we report also matches prior estimates for the electron-bunch emittance in the plasma, which were based on a combination of betatron-emission measurements and analytical models^27^,^28^,^36^. The precision of the technique we demonstrate can be further improved by extracting more information out of the angular-resolved x-ray spectrum, as well as utilizing the x-ray spatial profile.

In summary, we used the novel technique of spectroscopic imaging of ICS x-rays to non-destructively measure the intrinsic normalized transverse emittance of the LWFA electron beam immediately at the exit of the accelerator structure. The measured emittance is comparable to the best conventional LINAC accelerators. Ultra-small transverse emittance (*ε*_*nt*_) and source-size (*σ*_*r*_) of the beam translate into ultra-high spatial coherence(*L*_*c*_ ∝ *σ*_*r*_/*ε*_*nt*_)^37^, which is critical for applications such as ultra-fast electron diffraction. Our results have direct implications for narrowband ICS x-ray sources where high-charge beams with low-emittance are needed to maximize x-ray brightness and limit energy spread^38^. The ultra-small transverse emittance of the electron beam allowed us to demonstrate record-low energy spread of the all-laser ICS x-rays (24%), which is comparable to the x-ray bandwidth from conventional RF-LINAC-based Compton sources. Our technique can also be used to study the emittance growth of LWFA electron beams as they propagate through plasma-vacuum interface, providing critical insights necessary to minimize emittance growth on the interface^39^, or to optimize the coupling between stages in multiple-staged LWFA or a conventional beam transport line^40^,^41^. Since the technique is single-shot and does not require scanning^26^,^29^, it is suitable for electron beams that have shot-to-shot instability. The technique of spectroscopic x-ray imaging also holds promise to provide robust signatures of nonlinear Thomson scattering^42^ and radiation reaction^43^ in future ultra-high-intensity laser experiments.

## Methods

### Laser system

High-energy pulses from the Ti:Sapphire laser system were split at the end of the amplification chain using a 80% reflecting (20% transmitting) beam splitter and were independently compressed using 2-grating pulse compressors. The 80% reflected beam was compressed to 34 fs and focused by a f/14 parabolic mirror to a 20 um (FWHM) Gaussian focal spot. An adaptive optic loop composed of a deformable mirror and a wavefront sensor was used to compensate the optical aberrations^44^. We also used an adaptive closed loop to compensate spectral phase distortion^45^. This beam with energy of 1.4 J was used to drive a laser wakefield by focusing onto a double-nozzle gas target^4^ at 2 mm height above the nozzle orifice. The 20% transmitted beam with 0.3 J energy was compressed to 40 fs and focused to a 20-um (FWHM) Gaussian focal spot by another f/14 parabolic mirror to normalized field strength a_0_ = 0.7. It was used to generate x-rays by scattering off the LWFA electron beam with the focus of the scattering pulse being located 1.5 mm after the end of the second gas nozzle.

### Laser wakefield electron acceleration

The first and second nozzles (both 0.5 × 2 mm slits) of the double-jet gas target^4^ were operated with 99/1 mixture of helium and nitrogen and pure helium, respectively. The nozzles were separated by 0.5-mm gap. Three-dimensional density profiles of the gas flow from the nozzle were measured offline with a Mach-Zander interferometer and reconstructed using the SIRT tomography algorithm^46^. Energy of the electron beams was controlled by the second gas jet density. We used a magnetic spectrometer to characterize the energy spectra of the laser-produced electron beams. The spectrometer consisted of a round magnet (0.8 T, 19 cm diameter) and charge-calibrated LANEX screen imaged by a 12-bit CCD. The LANEX was located outside of the x-ray beam path to allow simultaneous measurement of both x-ray and electron beams. For the energy range reported in this work, the spectrometer had a resolution of 2%.

### Spectroscopic x-ray imaging

The energy spectrum of the ICS x-rays was measured using the HEXITEC spectroscopic imaging detector^47^. This system contains an energy-resolving Application-Specific Integrated Circuit (ASIC) which is bump-bonded directly to a 1-mm thick cadmium telluride (CdTe) detector. The output signal from each pixel is directly proportional to the energy deposited by an x-ray absorbed in the CdTe crystal. The detector consists of an array of 80 × 80 pixels on a 250 μm pitch with a total area of 20.35 mm × 20.45 mm. The position and precise energy deposited by each absorbed x-ray is recorded for each 100 μs frame. The recorded data is corrected for charge sharing events where x-rays that share energy between two or more pixels are removed. Without correction these shared events would incorrectly appear as multiple lower energy photons^48^. Each pixel is able to resolve x-ray energies with a resolution of 0.8 keV at 59.5 keV and 1.5 keV at 141 keV^47^. We carefully shielded the detector from background radiation comprised primarily of secondary radiation produced by electrons scattered off the chamber walls and the electron beam dump. The shielding was based on MCNP simulations of the background radiation in the experimental area. As a result, we were able to take measurements with negligible background. The distance between the detector and the source was chosen to be ~3 m in order to ensure that the photon flux on the detector was <0.1 photon/pixel. Based on Poisson statistics, we computed that the probability of 2 photons hitting a pixel is <1% and therefore pile-up effects could be neglected. The detector was aligned with the drive laser beam. X-ray beam position on the detector was determined by analyzing the spatially-resolved x-ray spectra.

### ICS modeling

In order to model the scattering process, we used the well-known result for the cross-section for Thomson scattering for a single electron (averaged over the azimuthal angle), given by
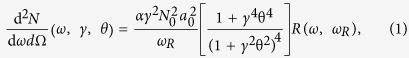
where *w*–frequency of the laser light, Ω–solid angle, *α*–fine structure constant, *γ* is the electron Lorentz factor, *N*_0_–number of laser oscillations the electron sees, and *a*_0_ = 0.85 × 10^−9^*λ[*μm](*I*[W/cm^2^])^1/2^–dimensionless laser strength parameter (*λ–*laser wavelength, *I*–intensity), *θ–*polar observation angle^49^. The resonance frequency *w*_*R*_ of the scattered light is given as
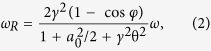
where *φ* is the collision angle between the electron and the laser pulse (170° in our geometry) and *w* is the laser pulse frequency. The resonance function *R*(*ω*, *ω*_*R*_)depends on the laser pulse shape. For long laser pulses (*N*_0_ ≫ 1, *N*_0_ ~ 10 for our experiment), *R*(*ω*, *ω*_*R*_) ∝ *δR*(*ω* − *ω*_*R*_) so that photons with specific energies are emitted along specific angles (not cones). In order to compute the scattered photon spectrum as a function of angle for the electron beams used in our experiment, equation (1) is integrated as follows:
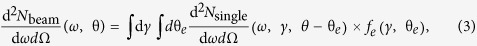
The quantity *f*_*e*_(*γ*, θ_*e*_)is the energy-angle distribution of the LWFA electron beam. The Supplementary Material provides an analysis of the range of validity of the model, and a cross-check with a fully relativistic 3D numerical model of ICS.

## Additional Information

**How to cite this article**: Golovin, G. *et al*. Intrinsic beam emittance of laser-accelerated electrons measured by x-ray spectroscopic imaging. *Sci. Rep.* **6**, 24622; doi: 10.1038/srep24622 (2016).

## Supplementary Material

Supplementary Information
