# Supplementary material for: Intrinsic beam emittance of laser-accelerated electrons measured by x-ray spectroscopic imaging
Source: Sci Rep. 2016 Apr 19;6:24622. doi: 10.1038/srep24622 (PMC4835856; doi:10.1038/srep24622)
Supplement: Supplementary Information [file srep24622-s1.pdf]

Intrinsic beam emittance of laser-accelerated electrons measured by x-ray spectroscopic imaging

G. Golovin[1], S. Banerjee[1], C. Liu[1], S. Chen[1], J. Zhang[1], B. Zhao[1], P. Zhang[1], M. Veale[2], M. Wilson[2], P. Seller[2], and D. Umstadter[1*]

Supplementary material

Range of validity for the simplified analytical model of ICS

The analysis used to determine the characteristics of the electron beam from the properties of the scattered x-rays includes the electron-beam and scattering-laser-pulse parameters, as well as the interaction geometry. In the regime of linear scattering ($a_0 \ll 1$), the spectral spread of the scattered x-rays is given by

$$\left.\frac{\Delta\omega_R}{\omega_R}\right|_{\Theta=0} \approx \sqrt{\left(\frac{\Delta\omega_0}{\omega_0}\right)^2 + \left(\frac{\lambda_0}{2\pi r_0}\right)^2 + \left(2\frac{\Delta\gamma}{\gamma_0}\right)^2 + \gamma_0{}^4\Delta\theta^4},$$

where head-on scattering is assumed.[1] The first two terms are contributions from the spectral bandwidth of the laser ($\Delta\omega_0$ is spectral width of the laser pulse, ω_0 is its central frequency), and the size of the laser focus (λ_0 is central wavelength, r_0 is focal spot radius). The third and the fourth term arise from the energy spread of the electron beam (γ_0 is the electron beam energy, $\Delta\gamma$ is the electron energy spread), and the angular divergence, $\Delta\theta$. For the experimental conditions applicable to our measurements [see Figure 2 caption in the main text, electron beam with a central energy of 65 MeV, energy spread of 9 MeV (FWHM), and angular divergence of 2.1 mrad], we obtain the following contributions to the energy spread: (i) laser bandwidth ~2.5%, (ii) size of the laser focus 0.02%, (iii) electron beam energy spread 27%, and (iv) electron beam divergence 7%; total bandwidth is 28% (experimentally measured value is 28±6%, see Figure 2 in the main text). Thus, the spatial and spectral contributions to the width of the x-ray spectrum from the laser pulse are negligible, as compared to those from the electron beam energy and angular spread. We therefore neglect laser pulse effects in our simplified theoretical model (Eqns. 1-3 in the main text), and consider only the effects due to the electron beam. The energy spread and divergence, for which electron beam effects become comparable to the laser pulse effects, are calculated to be 1.3% and 1.3 mrad, respectively (for 0.8 MeV energy spread for 61 MeV electron beam). We use these limits when inferring electron beam parameters based on the measurement of the x-ray spectrum.

[1] *Department of Physics and Astronomy, University of Nebraska, Lincoln NE 68588, USA*
[2] *Science and Technology Facilities Council, Rutherford Appleton Laboratory, Harwell Science & Innovation Campus, Didcot OX11 0QX, UK*
* e-mail: donald.umstadter@unl.edu

Comparison of the simplified analytical model and 3D numerical model

To verify the simplified analytical model of ICS (Eqns. 1-3 in the main text), we tested it against a fully relativistic 3D numerical model.[2, 3, 4] ICS was simulated with an intense Gaussian laser pulse (λ_0=800 nm, $I_0=9\text{x}10^{16}$ W/cm^2, 90 fs pulse duration, 12 µm FWHM focus size) counter-propagating with an electron beam (100 MeV central energy, 24% FWHM energy spread, 12 fs FWHM bunch duration, 2.4 mrad FWHM angular divergence, and 12 µm FWHM transverse spatial width). The full 3D numerical model used all these parameters to accurately describe the beams and took into account geometrical overlap between them. Since perfect overlap and linear interaction are assumed in the simplified analytical model, it requires only the following parameters: electron beam central energy, energy spread, angular divergence, and laser beam central wavelength. Supplementary figure 1 shows the comparison between these models for 3 polar angles: 0 mrad (on-axis scattering), 1.6 mrad, and 3.2 mrad. The spectra were normalized, since we are only interested in spectral shapes and not photon number predictions. As can be seen, the simplified analytical model shows very close match to the results of the 3D numerical model. Such close matching validates the simplifications we used.

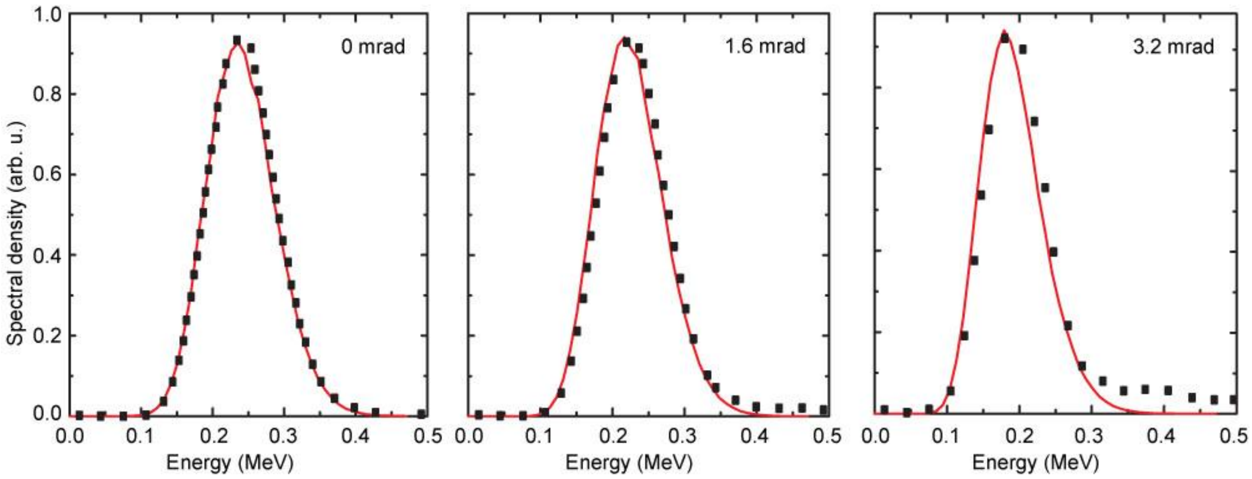

Supplementary figure 1. Normalized angular-resolved x-ray spectrum for ICS of a Gaussian laser (pulse λ_0=800 nm, $I_0=9\text{x}10^{16}$ W/cm^2, 90 fs pulse duration, and 12 µm FWHM focus size), counterpropagating with an electron beam (100 MeV central energy, 24% FWHM energy spread, 12 fs FWHM bunch duration, 2.4 mrad FWHM angular divergence, and 12 µm FWHM transverse spatial width). Black boxes – fully relativistic 3D numerical model,[2] red line – simplified analytical model (equations 1-3 in the Methods section of the main text). The spectrum is calculated for 3 polar angles - 0 mrad (on-axis scattering), 1.6 mrad, and 3.2 mrad.

Off-axis ICS spectroscopy for high-precision emittance measurements

We performed two sets of x-ray spectroscopic imaging measurements, single- and multiple-shot. The single-shot one is presented in the main text. The multiple-shot one is shown here to demonstrate how on-axis and off-axis ICS x-ray measurements affect the uncertainty of the electron-beam parameter determination.

For the multiple-shot measurement, we collected 14 shots. Stable accelerator operation over many shots was obtained at the cost of higher energy spread, as compared to the single-shot case. The charge of the electron beam varied from 2 – 12 pC. The x-ray spectrum accumulated for this sequence is shown on Supplementary figure 2(a) as a function of polar angle θ. The computed mean energy and energy spread as a function of polar angle are obtained using Gaussian fitting procedure to the x-ray spectrum and are shown in Supplementary figure 2(b) and (c), respectively.

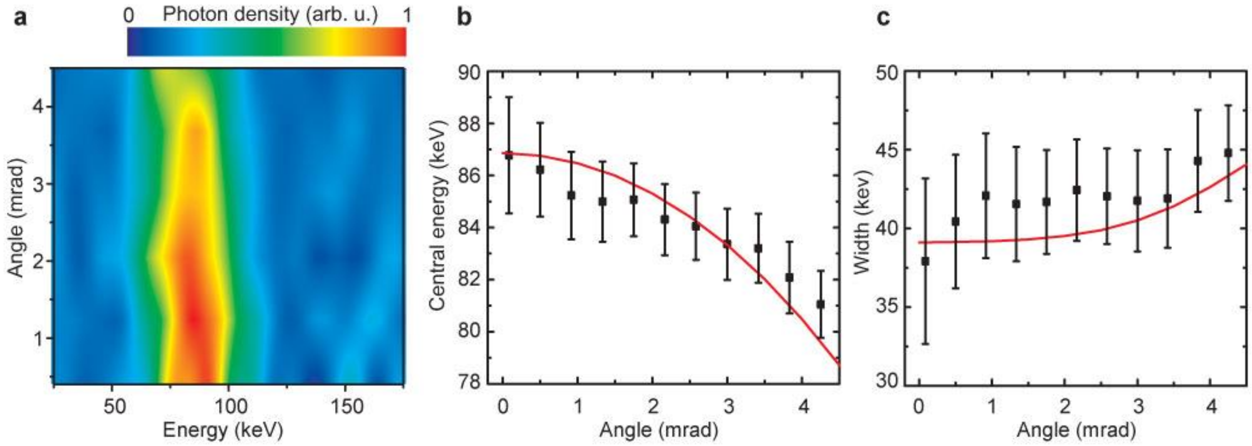

Supplementary figure 2. Angular-resolved x-ray spectrum measured using spectroscopic x-ray imaging for the multiple-shot measurement. (a) X-ray photon spectrum as a function of polar angle. (b) Mean energy of the x-rays as a function of polar angle. (c) Dependence of the spectral width (FWHM) on the polar angle. Averaged electron beam parameters were: central energy - 61±1 MeV, energy spread - 12±1 MeV (FWHM), and divergence - of 6±1 mrad (FWHM). Red curves on (b, c) show simulated dependences based on electron beam parameters, estimated from angular-resolved x-ray spectrum; central energy – 62.2 MeV, energy spread (FWHM) – 12.4 MeV, and divergence (FWHM) – 7.3 mrad.

We then used the angular-resolved spectral measurements to determine the parameters of the electron beam. To do so, we performed ICS simulations, varying three parameters of the electron beam: central energy, energy spread, and divergence. For each combination of these parameters (or for each point in 3D parameter space), we obtained an angle-resolved simulated x-ray spectrum. We described these spectra with three quantities: on-axis central energy, off-axis (θ = 4 mrad) central energy, and on-axis energy spread. We were then able to obtain electron beam parameters, such that the computed and measured x-ray parameters coincide. For the multiple-shot measurement, the resultant electron beam parameters, inferred from x-ray measurements, are shown in Supplementary figure 3. This figure illustrates the importance of the off-axis spectral x-ray measurements. If only on-axis x-ray spectral information is used (namely, on-axis central energy and energy spread), the uncertainty in 3-D electron-beam parameter space is rather large (see Supplementary figure 3(a), dark blue volume). When off-axis x-ray spectral information is added (namely, off-axis central energy), the uncertainty space shrinks significantly (see Supplementary figure 3(a), magenta volume).

The magenta volume on Supplementary figure 3(a) shows all possible combinations of electron beam parameters (central energy, energy spread, and divergence) which would result in x-ray

parameters we measured, and therefore provides strong limits to the associated uncertainties. The results are as follows: central energy – 62.2±1.6 MeV, energy spread (FWHM) – 12.4±2.9 MeV, and divergence (FWHM) – 7.3±2.3 mrad. Modelled angular dependencies of x-ray central energy and spread for these parameters are shown on Supplementary figure 2(b, c) as red curves. These parameters match well with the measurements obtained with the magnetic spectrometer: central energy – 61±1 MeV, energy spread – 12±1 MeV, and divergence – 6±1 mrad. It is important to note that, due to pointing fluctuation of ICS x-rays, the multiple-shot measurement resulted in an overestimated value for the electron beam divergence.

Supplementary figure 3(b) illustrates the impact of individual x-ray spectral quantities on the uncertainty of electron beam parameter determination. For ease of visualization, it shows only a slice of 3-D electron beam parameter space - energy spread and divergence, with central energy fixed at 62 MeV. Geometrically, it corresponds to the plane indicate by the black square in Supplementary figure 3(a). Light blue (medium blue, pink) area corresponds to combinations of electron beam parameters which would result in ICS x-rays with the measured on-axis central energy (off-axis central energy, on-axis energy spread). The width of the bands is related to the x-ray measurement uncertainty: the better the x-ray measurement, the narrower the bands should be. The dark blue area is the intersection of individual measurements performed with on-axis ICS x-ray central energy and energy spread; it is a cross-section of the dark blue volume shown in Supplementary figure *3*(a). The magenta area corresponds to the intersection of all "individual" measurements (both on-axis and off-axis); it is also a cross-section of the magenta volume shown in Supplementary figure 3(a).

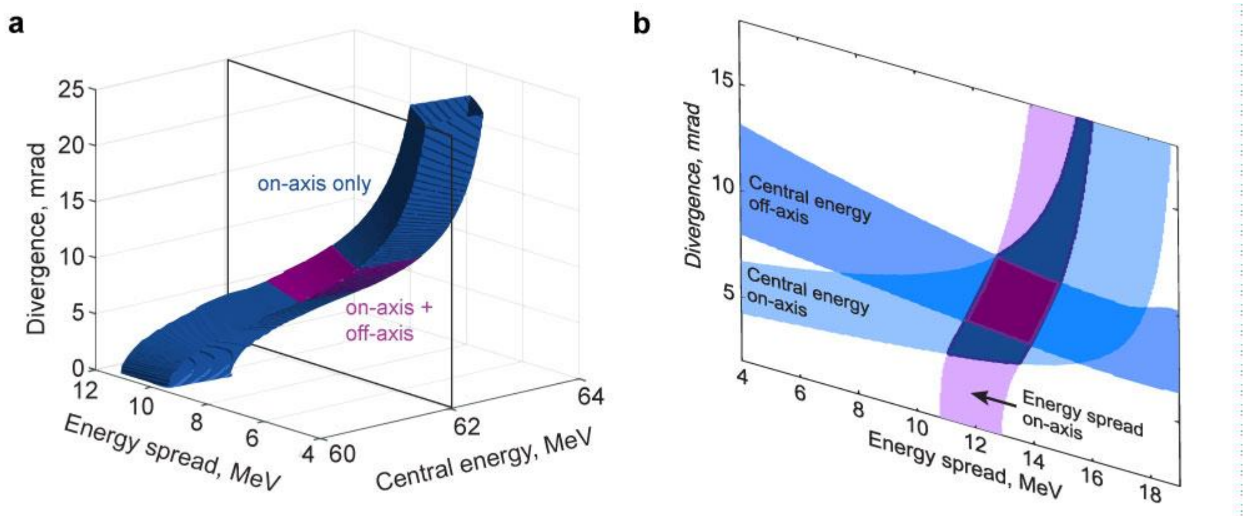

Supplementary figure 3. Determination of the electron beam parameters based on the on-axis and off-axis (θ=4 mrad) ICS measurements, multiple-shot measurement. a) Divergence, energy spread, and central energy form a 3D parameter space of the electron beam. By measuring ICS spectrum on-axis only (with the measurement uncertainty shown on Supplementary figure *2*(b) and (c)), one can estimate the electron beam parameters with uncertainty, shown as blue volume. Once off-axis scattering spectrum is also measured, the uncertainty in electron beam parameters goes down (magenta volume). b) The parameter space cross-section, (corresponding to the black box on figure a). Individual contribution of central energy and energy spread of the scattered x-rays are shown.

Angular-resolved x-ray spectral measurement

This section expands on the angular-resolved x-ray spectral measurement, shown in Figure 2 of the main text. The shot we used for this measurement resulted in 213 photons detected on the CdTe detector. To measure a spectrum for a particular polar angle, we chose a ring area around that particular angle on the CdTe image. The width of this area was 2.5 mrad (30 pixels). All photons detected within that area were then used to form the spectrum. Due to this procedure, every data point on figure 2 (main text) represents the spectrum within the polar angle area of [Θ-1.25 mrad; Θ+1.25 mrad], where Θ – polar angle of a particular data point. The same is true for Supplementary figure 4a. The width (2.5 mrad) was chosen to collect enough photons for meaningful spectral measurements for each polar angle. Minimal photon number (collected for Θ = 0 mrad) was 30, this number increased (with geometrical area of the rings) up to 90 (for Θ = 4 mrad). The results are shown on Supplementary figure 4a.

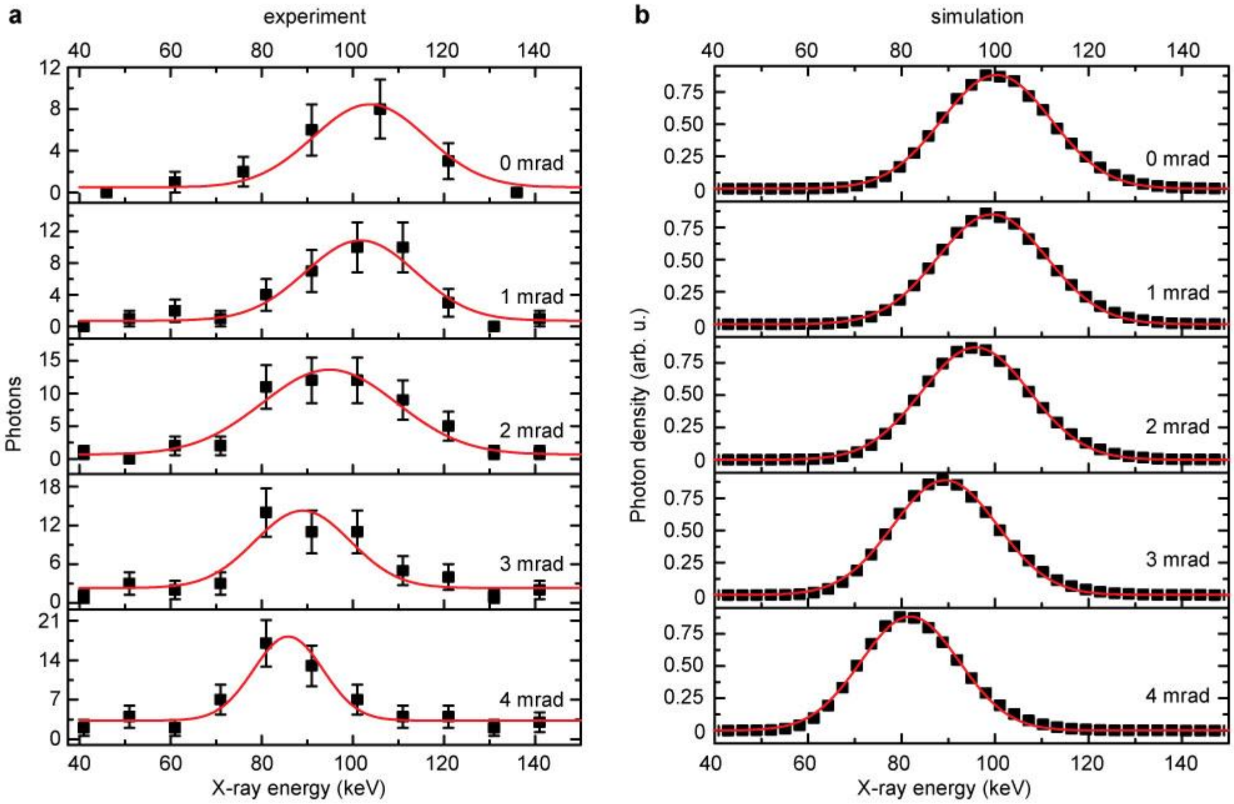

Supplementary figure 4. Experimentally measured (a) and simulated (b) angular-resolved x-ray spectrum as a function of polar angle. Experimentally measured electron beam parameters: central energy - 65±1 MeV, energy spread (FWHM) - 8.9±0.1 MeV, divergence (FWHM) - 4.1±0.4 mrad (on the LANEX screen). The simulation performed for the following electron beam parameters: central energy – 65 MeV, energy spread (FWHM) – 8.9 MeV, divergence (FWHM) – 2.1 mrad. Red curves show Gaussian fits of the data. Error bars calculated according Poisson statistics.

The photon number (30-90) was quite small to measure fine features of the spectrum. Fortunately, the task was easier since inverse-Compton x-ray spectrum (with electron beam and

scattering laser pulse parameters relevant to our experiment) is very close to Gauss function, as it follows from the simulation we performed (see Supplementary figure 4b). The simulated data are fitted with Gaussian functions, the quality-of-fit parameter R^2>0.99, indicating very good fits. Assuming Gaussian spectral shapes, we therefore used detected photon data to extract only 2 parameters out of each spectrum, namely peak position and width. With higher photon number we would be able to extract even more information (for example, peak position, width, and skewness), which would decrease the uncertainty of our measurement.

The relationship of Supplementary figure 4 and figure 2 in the main text is as follows. Supplementary figure 4a (points) and figure 2a show the same data. The positions of the centers and widths of the Gaussian fits (red curves) on the Supplementary figure 4a are shown as points on figure 2b and c. The positions of centers and width of the Gaussian fits (red curves) on the Supplementary figure 4b are shown as red lines on figure 2b and c.

Simulations of the electron beam phase space evolution due to space charge forces

In this section, we provide additional aspects of the change in the electron beam phase space after it exits the accelerator on account of the internal space charge force. The role of this force in degrading the emittance of the electron beam has been discussed in the manuscript. In the following we provide further details of this process, and also consider its effect on other parameters of the electron beam that are relevant to the application of these high-charge, ultrashort beams for the generation of high-brightness x-rays, studies of femtosecond dynamics, and injection into secondary accelerating structures.

After the beam exits the plasma, the focusing forces are no longer present. As a result, the space charge force will tend to increase both the transverse and longitudinal velocity spreads. The latter is smaller on account of the electron beam being highly relativistic. The transverse extent of the beam is determined by the initial size at the exit, and the intrinsic transverse momentum spread. The latter is enhanced on account of the space charge and leads to a more rapid increase in the size of the electron beam as it propagates. In case of the longitudinal dimension, the finite energy spread will lead to an increase in the temporal duration of the beam, and this effect is further amplified under the effect of space charge forces.

For these simulations, we use the following parameters: (i) energy $\gamma = 122,$ (ii) energy spread $\Delta\gamma = 10,$ (iii) transverse velocity $\gamma\beta_x = \gamma\beta_y \to 2.1$ mrad (iv) source size $\sigma_r = 4$ microns, (v) pulse duration $\tau = 10$ fs (FWHM), and (vi) beam charge $q = 10$ pC. The evolution of the beam is then computed over a range of 1 m. Close to the source, the beam evolution is sampled in 250 – 500 micron steps, and this is increased to several mm further out where the rate of change is more gradual. The change in the transverse momentum spread of the beam is shown in Supplementary figure 5(a). The blue curve denotes the case where space charge forces are taken into account, while the red curve is for the case when space charge is neglected. As expected, the transverse velocity spread increases on account of the effect of the space charge. This increase in transverse

velocity results in the change in the divergence angle of the beam shown in the paper. As can be seen, even a small change in the transverse spread leads to a significant increase in the divergence of the beam at the early times when the size of the beam is small. We also computed the change in the longitudinal energy distribution. The change in the initial energy spread of the beam is small ~2% ($\Delta\gamma : 10 \rightarrow 10.1$).

It is also of interest to determine the change in the beam size due to space-charge-driven dynamics, as well the effect, if any, on the temporal duration of the electron beam. For the case considered here, the results are shown in Supplementary figure 5(b). Unlike the case for the transverse velocity distribution, no significant difference is observed in the far-field for the beam size and pulse duration. The 4-fold increase in pulse duration is large, driven by the dispersion of the electron beam on account of the initial energy spread. These results show that, for studies where ultrashort electron beams are required, the useful distance from the source is <10 cm.

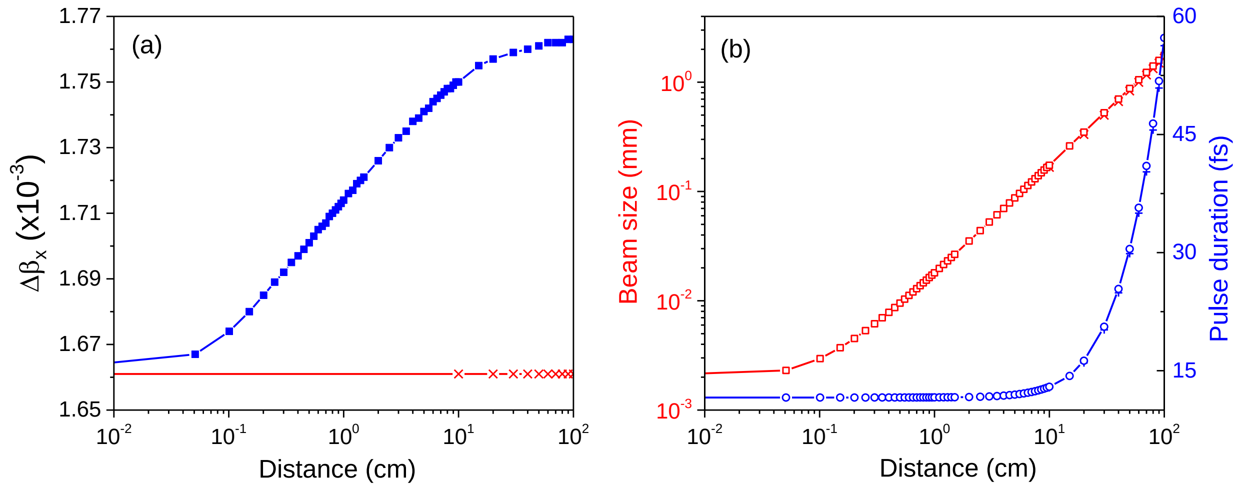

Supplementary figure 5: (a) Change in the transverse velocity of the beam as it propagates after exiting the plasma. The blue curve shows this change when space charge forces are taken into account. The blue squares correspond to the points at which the transverse velocity spread is evaluated. The red curve is for the case when space charge is neglected. (b) Evolution of the transverse electron beam size and pulse duration as the beam propagates after exiting the plasma. The initial size of the beam is chosen to be consistent with our measurements using the knife edge technique and the pulse duration is assumed based on prior measurements of the duration of a laser wakefield accelerated beam. In this case, no significant difference is observed between the cases where space charge is taken into account and where it is neglected in the far field. For the beam size, the red squares are for the case when space charge is taken into account, and the red crosses are for the case when space charge is neglected. For the pulse duration, blue circles correspond to space charge driven beam evolution, and blue plus signs are for the case in which space charge is ignored.

References

1. Brown, W. J. & Hartemann, F. V. Three-dimensional time and frequency-domain theory of femtosecond x-ray pulse generation through Thomson scattering. *Physical Review Special Topics-Accelerators and Beams* **7**, 060703 (2004).
2. Ghebregziabher, I., Shadwick, B. A. & Umstadter, D. Spectral bandwidth reduction of Thomson scattered light by pulse chirping. *Phys. Rev. ST Accel. Beams* **16**, 030705 (2013).
3. Chen, S. *et al*. MeV-Energy X Rays from Inverse Compton Scattering with Laser-Wakefield Accelerated Electrons. *Phys. Rev. Lett.* **110**, 155003 (2013).
4. Powers, N. D. *et al*. Quasi-monoenergetic and tunable X-rays from a laser-driven Compton light source. *Nature Photonics* **8**, 29-32 (2014).
